# Healthcare-Seeking Behaviour Due to Cough in Finnish Elderly: Too Much and Too Little

**DOI:** 10.1007/s00408-023-00595-w

**Published:** 2023-01-26

**Authors:** Johanna Tuulikki Kaulamo, Anne Marika Lätti, Heikki Olavi Koskela

**Affiliations:** 1 School of Medicine, Institute of Clinical Sciences, Faculty of Health Sciences, University of Eastern Finland, Yliopistonranta 1, 70210 Kuopio, Finland; 2 Unit for Medicine and Clinical Research, Pulmonary Division, Kuopio University Hospital, Kuopio, Finland; 3 Mehiläinen Terveyspalvelut Oy, Healthcare Services for Prisoners, Kuopio, Finland

**Keywords:** Chronic cough, Health-seeking behaviour, Asthma, Bronchiectasis, Quality-of-life

## Abstract

**Introduction:**

Cough-related healthcare-seeking has not been studied specifically in the elderly, although chronic cough is most prevalent among them. We studied the frequencies and predictors of any (≥ 1) and repeated (≥ 3) doctor’s visits due to any cough episode during the past year, and due to the current cough episode.

**Methods:**

This was a cross-sectional email survey among a Finnish community-based elderly population. Participants with current cough and age ≥ 64 years were included in the analyses (*n* = 1109).

**Results:**

The proportions of participants with ≥ 1 and ≥ 3 cough-related doctor’s visits during the past year were 25.9% and 7.1%, respectively. Repeated visitors accounted for 55.9% of the visits during the past year. These visits first increased with cough duration but decreased after 5 years. In the multivariate analysis, bronchiectasis [aOR 3.22 (CI95% 1.08–9.58)], asthma [2.62 (1.56–4.40)], chronic sputum production [1.61 (0.94–2.76)], low self-assessed health status [1.40 (1.04–1.88)] and Leicester Cough Questionnaire total score [1.34 per tertile (1.10–1.62)] predicted repeated cough-related doctor’s visits during the past year. The proportions of ≥ 1 and ≥ 3 doctor’s visits due to current cough were 31.8% and 15.5%, respectively. Among participants with current chronic cough, 60.1% had not visited a doctor.

**Conclusion:**

A minority of participants accounted for most of the cough-related doctor’s visits during the past year, whereas most participants with chronic cough had never sought medical help for it. The heavy healthcare users were not those with the longest cough episodes. Repeated visitors due to cough were characterised by chronic phlegmy respiratory conditions, and quality-of-life impairment.

## Introduction

Cough is the most common patient-reported reason to visit a doctor [1], causing a high demand for healthcare services. Early detection of life-threatening diseases necessitates keeping a low threshold to primary care evaluation. However, chronic cough can remain refractory to treatment or unexplained. This may leave the patient with an impaired cough-related quality-of-life [2, 3] and predisposed to repeated doctor’s visits, which add to the high socioeconomic burden of cough.

There is little data on healthcare-seeking behaviour due to cough in western countries. In our previous study, impaired cough-related quality-of-life and asthma were the strongest predictors of repeated doctor’s visits during the previous year in working age adults with current cough [4]. In that study, most of the cough-related doctor’s visits were accounted by a minority of participants. In general, older age, female gender, low socioeconomic status and multimorbidity are associated with high healthcare costs [5]. The elderly is most burdened by chronic cough in prevalence and in number of cough background disorders [6]. However, cough-related doctor’s visits and the characteristics of repeated visitors due to cough have not been studied specifically in the elderly. This data could be useful in using healthcare resources more cost-effectively.

This study was an email survey among the members of a nationwide pensioner organisation in Finland. The first outcome was cough-related doctor’s visits during the past year, which could include visits due to several separate cough episodes. The second outcome was doctor’s visits due to the current cough episode, which could have lasted for several years.

## Methods

### Population

This was a cross-sectional email survey among the members of the Finnish Pensioners` Federation (26,205 members who had an email address, with mean age of 72.7 years, and 63.5% females). The invitation, including information about the study, and the questionnaire were sent in April 2021. A reminder message was sent 2 weeks later. The responses were collected electronically. The decision to respond was considered as an informed consent. The study was approved by the Ethics Committee of Kuopio University Hospital (289/2015). Permission to conduct the study was obtained from the Finnish Pensioners` Federation. Patients were not involved in the design or conduct of this study.

### Questionnaire

There were 62 questions about age, socioeconomic status, smoking, alcohol consumption, recently experienced symptoms, general health, disorders diagnosed by a doctor, medications, and number of cough-related doctor’s visits during the past year. Asthma, chronic rhinosinusitis, gastro-esophageal reflux disease (GERD) and obstructive sleep apnea (OSA) were inquired by questions recommended for epidemiological studies [7–11]. Depressive symptoms were asked using the Patient Health Questionnaire-2 (PHQ-2) [12]. The 24 additional questions about current cough included details about cough frequency and duration, the Leicester Cough Questionnaire (LCQ) to investigate the cough-related quality-of-life, and number of doctor’s visits due to current cough. The questionnaire has been used in our previous email survey [13], and it was modified for this study.

### Definitions

Current cough was defined as presence of cough within 2 weeks. Acute, subacute, and chronic cough were defined by duration of current cough (< 3 weeks, 3–8 weeks, and > 8 weeks, respectively)[14]. Current asthma was defined as doctor’s diagnosis of asthma at any age and wheezing during the past year [7]. Chronic rhinosinusitis was present if there was either nasal blockage or discharge (anterior or posterior nasal drip), and either reduction/loss of smell or facial pain/pressure for at least 3 months during the past year [8].

GERD was defined as presence of heartburn or regurgitation at least once a week in the last 3 months [9]. OSA was defined as presence of ≥ 2 of the following features: Loud snoring, daytime tiredness, observed apneas and arterial hypertension [10, 11]. Somatic sum was defined as the sum (0–15) of experienced symptoms during the last month, excluding respiratory symptoms. Disorder sum (0–19) was defined as the number of medical conditions diagnosed by a doctor, excluding background disorders of chronic cough [6]. Depressive symptoms were present if PHQ-2 score was ≥ 3 [12]. Family history of chronic cough was defined as cough lasting > 8 weeks in parents or siblings. Trigger sum (0–15) was defined as the sum of external cough-triggering factors. Allergy was defined as self-reported allergy to animals, pollens, or food. Chronic sputum production was defined as cough with phlegm on most days or nights for ≥ 3 months of the year [15].

#### The Outcome Variables

The main outcomes were any (≥ 1) cough-related doctor’s visit during the past year, repeated (≥ 3) cough-related doctor’s visits during the past year, any (≥ 1) doctor’s visit due to current cough, and repeated (≥ 3) doctor’s visits due to current cough. Cough-related doctor’s visits during the past year included all visits within the previous year that were due to all cough episodes, whereas doctor’s visits due to current cough included only visits during the current cough episode. The definition of repeated doctor’s visits as ≥ 3 is consistent with our previous work [4].

### Statistical Analysis

Descriptive data are shown as means and 95% confidence intervals. Chi-squared test, Mann–Whitney *U*-test and Kruskal–Wallis test was applied when appropriate. Bonferroni correction was applied for multiple comparisons. Bivariate association of the main outcomes and the following variables were analysed: Age, gender, body mass index, current smoking, ever smoking, alcohol consumption, years of education, income level, pet ownership, moisture damage exposure, acetylsalicylic acid intolerance, self-assessed heath status, family history of chronic cough, symptom sum, disorder sum, depressive symptoms, allergy, chronic obstructive pulmonary disease, chronic sputum production, bronchiectasis, pulmonary fibrosis, tuberculosis, sarcoidosis, current asthma, chronic rhinosinusitis, GERD, OSA, duration of current cough, LCQ total score (LCQt), LCQ domains, presence of any cough trigger, presence of a chemical cough trigger, and trigger sum. The variables were chosen in the multivariate analyses based on plausible association with doctor’s visits due to cough, prevalence of ≥ 2% in the study population regarding the etiological variables, and at least a suggestive association (*p* < 0.1) with the outcome variable. From the variables with strong interrelationships (symptom sum, disorder sum and self-assessed health status), one variable with the strongest bivariate association with the main outcome was included in the multivariate analysis. From the LCQ scores, only LCQt was included in the analyses because of strong interrelationships with the domains. The multivariate analyses were conducted using binary logistic regression with a backward directed stepwise process. A *p*-value < 0.05 was considered statistically significant, but suggestive associations (*p* < 0.1) are also presented. The analyses were conducted using SPSS v.27.

## Results

**Fig. 1:**
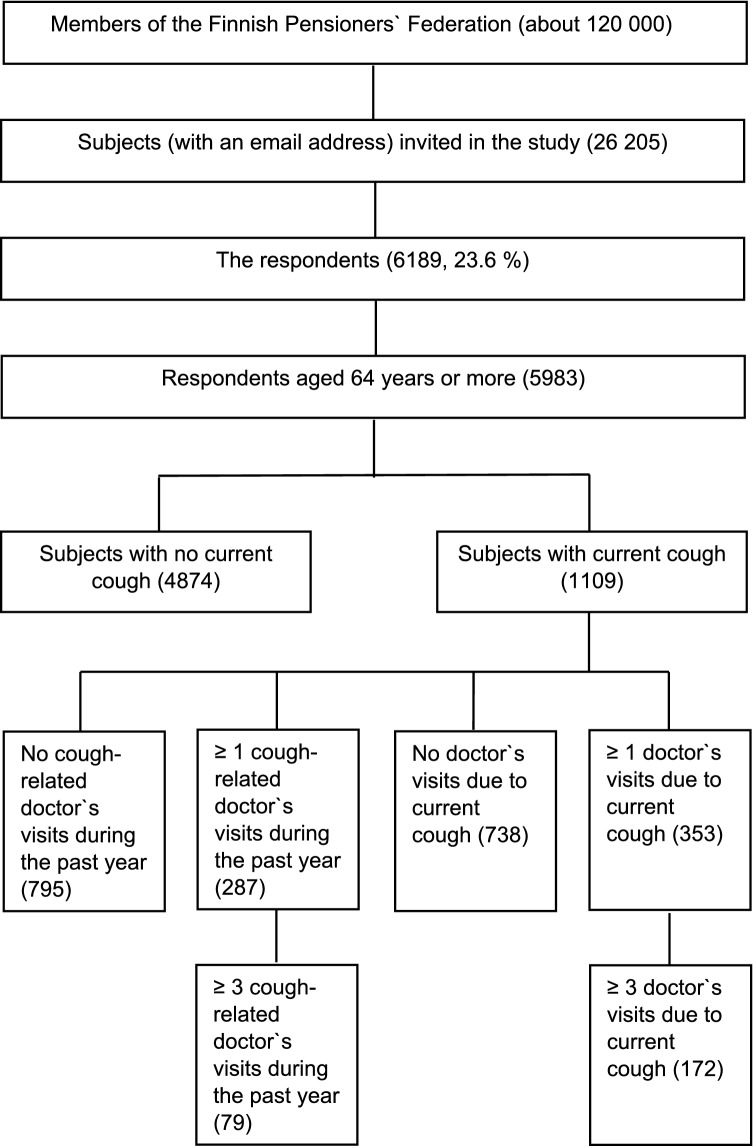
Flow chart of the study population

**Fig. 2:**
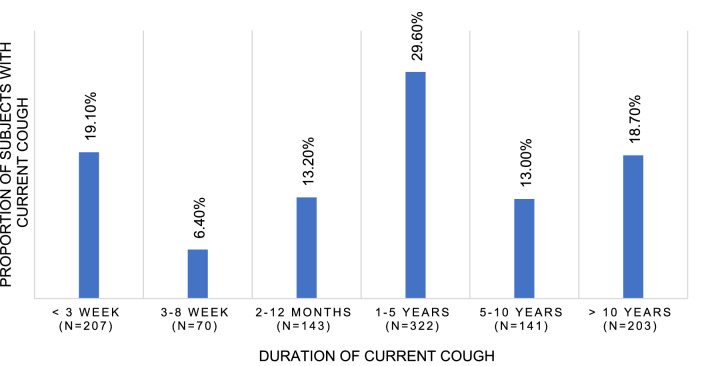
Distribution of cough duration among the study subjects (*n* = 1109). 23 subjects could not define the cough duration

The response rate was 23.6% (*n* = 6189, mean age 72.2 (5.5) years, 66.4% female). The proportion of missing values was < 2.5%, except for the questions about family income (2.9%) and OSA (3.1–3.7%). The participants with current cough and age ≥ 64 years were included in the analyses (*n* = 1109, mean age 72.9 (5.3) years, 67.7% female) (Fig. 1). As the distribution of cough duration shows (Fig. 2), most participants had chronic cough of > 1 year of duration. The doctor’s visits in acute, subacute and chronic cough are presented in Table 1.

**Table 1:** Doctor's visits according to cough status among the study subjects (*n* = 1109)

Doctor's visits	Current acute cough (*n* = 207)	Current subacute cough (*n* = 70)	Current chronic cough (*n* = 809)
≥ 1 cough-related visits during the past year^a^	16.3%	23.2%	29.8%***
≥ 3 cough-related visits during the past year^a^	2.5%	5.8%	8.8%**
≥ 1 visits due to the current cough episode	9.3%	17.4%	39.9%***^+++^
≥ 3 visits due to the current cough episode	2.0%	4.3%	20.5%***^++^

23 subjects could not define the cough duration

Between acute and chronic cough: *p < 0.05, **p < 0.01, ***p < 0.001

Between subacute and chronic cough: ^+^*p* < 0.05, ^++^*p* < 0.01, ^+++^*p* < 0.001

There were no statistically significant differences between acute and subacute cough

^a^Due to any cough episode

### Number of Cough-Related Doctor’s Visits During the Past Year

**Fig. 3:**
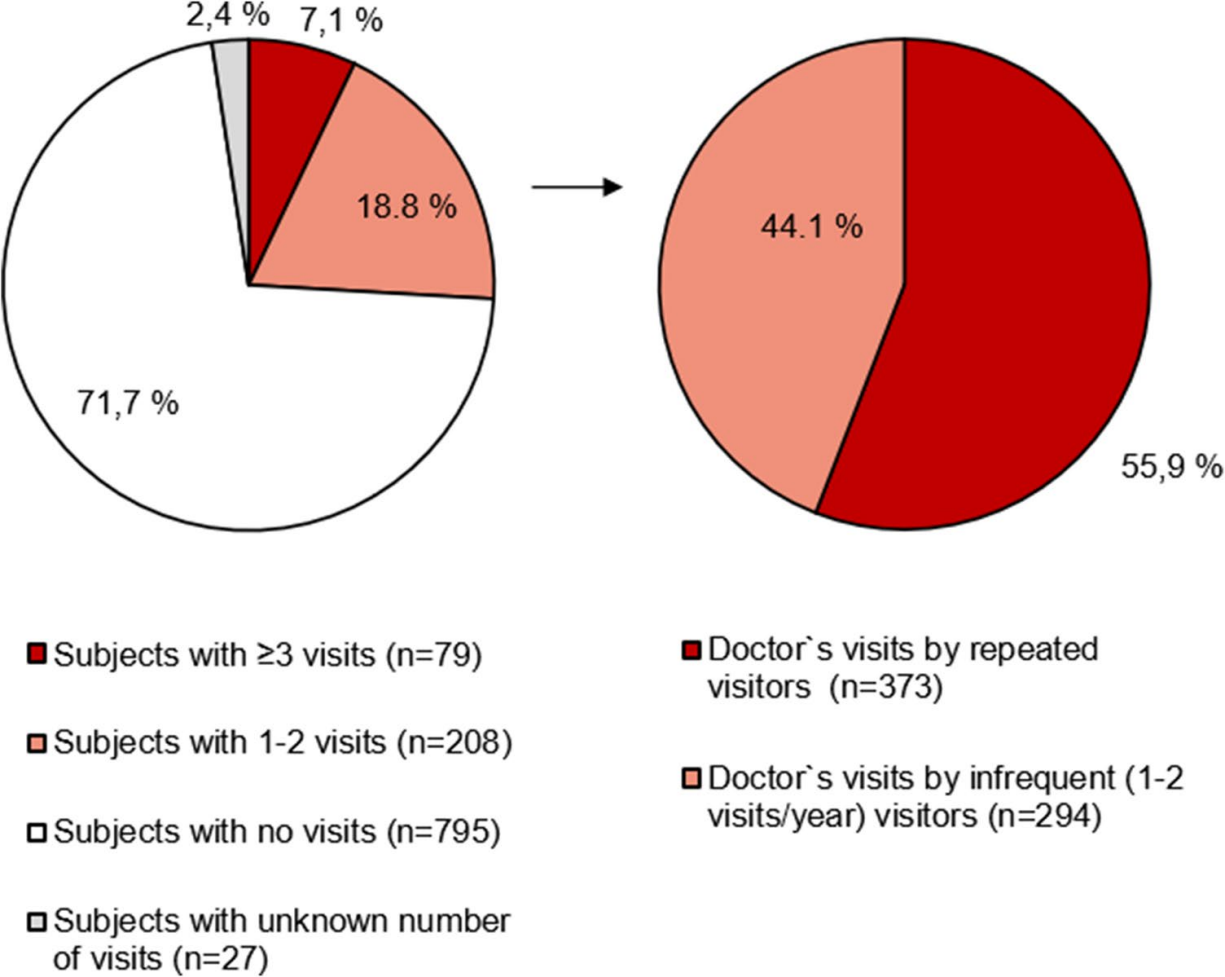
Distribution of the subjects by the number of cough-related doctor’s visits during the past year, followed by distribution of repeated and infrequent cough-related doctor’s visits during the past year

**Fig. 4:**
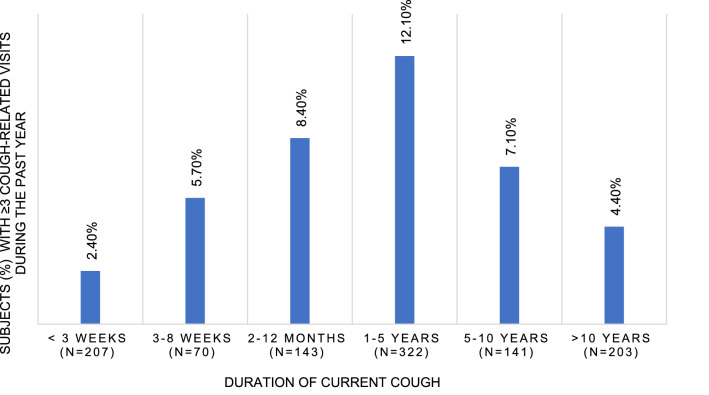
Percentage of subjects with repeated (≥ 3) cough-related doctor’s visits during the past year, according to duration of current cough

Among the 1109 participants, there were 667 *cough-related doctor’s visits during the past year*. There were 287 (25.9%) participants who had visited a doctor at least once, and 79 (7.1%) repeatedly (Table 2). Those with repeated visits accounted for majority (55.9%) of the yearly visits (Fig. 3). As seen in Fig. 4, the percentage of participants with repeated visits increased until the duration of current cough reached 1–5 years and decreased after that.

**Table 2:** Characteristics of subjects with no cough-related doctor’s visits during the past year, and their bivariate associations with subjects with ≥ 1 visits. Subjects with ≥ 3 doctor’s visits are compared to those with 0–2 doctor’s visits

Characteristic	No cough-related doctor’s visits during the past year (*n* = 795)	≥ 1 cough-related visits during the past year (*n* = 287)	≥ 3 cough-related visits during the past year (*n* = 79)
Age, years	72.6 (72.3–73.0)	73.4 (72.8–74.0)*	72.9 (71.7–74.0)
Female gender, %	68.4	65.2	68.4
Body mass index, kg/m^2^	27.7 (27.4–28.0)	28.2 (27.6–28.9)	28.3 (26.8–29.8)
Education years	12.77 (12.51–13.03)	12.61 (12.20–13.03)	12.70 (11.88–13.53)
Income level^a^	2.36 (2.31–2.42)	2.34 (2.26–2.43)	2.23 (2.08–2.39)
Current smoking, %	2.4	1.4	1.3
Alcohol consumption, doses per week	3.2 (2.9–3.6)	3.7 (3.0–4.3)	4.3 (2.7–5.9)
Depressive symptoms, %	7.4	9.6	10.4
Self-assessed health status^b^	2.47 (2.41–2.53)	2.74 (2.64–2.83)***	2.87 (2.67–3.07)**
Symptom sum	2.74 (2.60–2.87)	2.96 (2.70–3.22)	2.89 (2.38–3.39)
Disorder sum	1.75 (1.66–1.85)	2.09 (1.92–2.25)***	2.19 (1.86–2.52)*
Family history of chronic cough, %	38.4	40.6	48.7
Allergy, %	14.5	16.7	24.1*
Current asthma, %	12.5	27.0***	36.4***
Bronchiectasis, %	1.3	3.8**	6.3**
Chronic obstructive pulmonary disease, %	2.3	5.9**	6.3
Chronic sputum production, %	47.2	67.6***	72.2***
Chronic rhinosinusitis, %	18.6	27.9***	31.6*
Gastro-esofageal reflux disease, %	27.0	30.4	30.8
Obstructive sleep apnea, %	45.0	48.6	52.6
Duration of current cough (median, range)	1–5 years (< 1 week to > 10 years)	1–5 years (< 1 week to > 10 years)	1–5 years (< 1 week to > 10 years)
Trigger sum	3.49 (3.28–3.70)	4.24 (3.81–4.67)*	4.68 (3.76–5.61)
Leicester cough questionnaire, total score	15.91 (15.73–16.10)	13.33 (12.96–13.70)***	12.06 (11.28–12.85)***

The figures are presented as percentages or means and 95% CI unless stated otherwise

*p < 0.05, **p < 0.01, ***p < 0.001

^a^Household income per year: (1) Under 15,000 e, (2) 15,000–40,000 e, (3) 40,000–70,000 e, (4) 70,000–120,000 e, (5) over 120,000 e

^b^Self-assessed health status: (1) Good, (2) Rather good, (3) Mediocre, (4) Rather poor, (5) Poor

### Predictors of Cough-Related Doctor’s Visits During the Past Year

The bivariate associations of any and repeated cough-related doctor’s visits during the past year are presented in Table 2. In the multivariate analysis, current asthma, low LCQt, chronic sputum production, and impaired self-assessed health status were associated with ≥ 1 cough-related doctor’s visits during the past year (Table 3). Bronchiectasis, asthma, chronic sputum production, low self-assessed health status and LCQt predicted ≥ 3 cough-related doctor’s visits during the past year in the multivariate analysis (Table 3). Duration of current cough was associated with neither of the outcomes.

**Table 3:** Characteristics associated with cough-related doctor’s visits during the past year among subjects with current cough (*n* = 1109). Multivariate analyses with adjusted ORs and 95% confidence intervals

≥ 1 cough-related doctor’s visit during the past year (n = 287)	≥ 3 cough-related doctor’s visit during the past year (*n* = 79)
Current asthma 1.86 (1.30–2.66)***	Bronchiectasis 3.22 (1.08–9.58)*
LCQ total score^a^ 1.78 (1.46–2.17)***	Current asthma 2.62 (1.56–4.40)***
Chronic sputum production 1.52 (1.11–2.07)**	Chronic sputum production 1.61 (0.94–2.76)
Self-assessed health status 1.19 (1.00–1.42)	Self-assessed health status 1.40 (1.04–1.88)*
	LCQ total score^a^ 1.34 (1.10–1.62)**

*p < 0.05, **p < 0.01, ***p < 0.001

^a^Leicester cough questionnaire total score in reversed tertiles (21.0–16.92, 16.91–14.35 and 14.34–3.00)

### Number of Doctor’s Visits Due to Current Cough

**Fig. 5:**
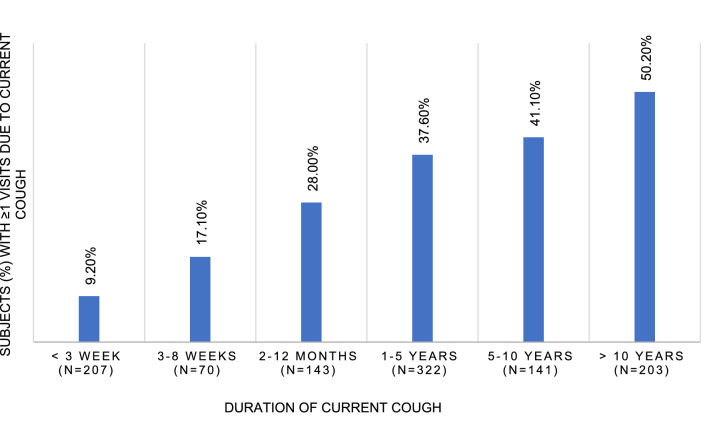
Percentage of subjects with any (≥ 1) doctor’s visit due to current cough, according to cough duration

Among the 1109 participants with current cough, 353 (31.8%) had visited a doctor due to current cough at least once, and 172 (15.5%) repeatedly (Table 4). Current chronic cough had prompted ≥ 1 doctor’s visits in 39.9% of the participants (Table 1). There were 1505 doctor’s visits due to current cough in total, and 82.6% of them were accounted by repeated visitors. As seen in Fig. 5, the percentage of subjects with ≥ 1 visits due to current cough increased linearly as the cough episode prolonged. However, half (49.8%) of the participants with cough persisting > 10 years had never visited a doctor due to current cough.

**Table 4:** Characteristics of the subjects with no doctor’s visits due to current cough, and their bivariate associations with subjects with ≥ 1 doctor's visits. Subjects with ≥ 3 doctor’s visits are compared to those with 0–2 visits

Characteristic	No doctor’s visits due to current cough (*n* = 738)	≥ 1 visit due to current cough (*n* = 353)	≥ 3 visits due to current cough (*n* = 172)
Age, years	73.0 (72.6–73.4)	72.6 (72.1–73.2)	72.6 (71.9–73.3)
Female gender, %	67.2	68.0	70.3
Body mass index, kg/m^2^	27.8 (27.5–28.1)	27.8 (27.2–28.4)	27.7 (26.8–28.6)
Education years	12.65 (12.39–12.92)	12.84 (12.44–13.24)	12.92 (12.32–13.52)
Income level^a^	2.34 (2.29–2.39)	2.37 (2.29–2.45)	2.34 (2.22–2.46)
Current smoking, %	2.6	1.1	0.0*
Alcohol consumption, doses per week	3.2 (2.8–3.6)	3.6 (3.0–4.3)	3.9 (2.8–5.0)
Depressive symptoms, %	7.7	8.4	6.5
Self-assessed health status^b^	2.52 (2.46–2.58)	2.60 (2.51–2.69)	2.70 (2.57–2.82)*
Symptom sum	2.77 (2.63–2.91)	2.81 (2.59–3.03)	2.83 (2.50–3.16)
Disorder sum	1.86 (1.76–1.96)	1.82 (1.67–1.96)	1.80 (1.60–2.01)
Family history of cc, %	38.6	37.6	44.1
Allergy, %	16.3	12.7	14.5
Current asthma, %	14.0	20.5**	26.5***
Bronchiectasis, %	1.6	3.1	4.7*
Chronic obstructive pulmonary disease, %	2.3	5.1*	4.7
Chronic sputum production, %	46.8	64.4***	69.4***
Chronic rhinosinusitis, %	19.0	24.4*	25.6
Gastro-esofageal reflux disease, %	26.9	29.7	28.1
Obstructive sleep apnea, %	45.2	48.4	49.1
Duration of current cough, (median, range)	1–5 years (< 1 week to > 10 years)	1–5 years (< 1 week to > 10 years)***	5–10 years (< 1 week to > 10 years)***
Trigger sum	3.49 (3.26–3.71)	3.96 (3.59–4.33)	4.36 (3.79–4.93)*
Leicester cough questionnaire total score	16.00 (15.82–16.20)	13.62 (13.30–13.95)***	12.96 (12.47–13.44)***

The figures are presented as percentages or means and 95% CI unless stated otherwise

*p < 0.05, **p < 0.01, ***p < 0.001

^a^Household income per year: (1) Under 15,000 e, (2) 15,000–40,000 e, (3) 40,000–70,000 e, (4) 70,000–120,000 e, (5) over 120,000 e

^b^Self-assessed health status: (1) Good, (2) Rather good, (3) Mediocre, (4) Rather poor, (5) Poor

### Predictors of Doctor’s Visits Due to Current Cough

The bivariate associations of any and repeated doctor’s visits due to current cough are presented in Table 4. Results of the multivariate analyses of doctor’s visits due to current cough are presented in Table 5. Cough-related quality-of-life impairment and cough prolongation predicted both any and repeated doctor’s visits due to current cough. Furthermore, current asthma and chronic sputum production increased the risk of repeated doctor’s visits.

**Table 5:** Characteristics associated with doctor’s visits due to current cough among participants with current cough (*n* = 1109). Multivariate analyses with adjusted ORs and 95% confidence intervals

≥ 1 doctor’s visits due to current cough (*n* = 353)	≥ 3 doctor’s visits due to current cough (*n* = 172)
LCQ total score^a^ 1.76 (1.49–2.10)***	Cough duration^b^ 1.75 (1.53–2.00)***
Cough duration^b^ 1.43 (1.32–1.56)***	Current asthma 1.71 (1.11–2.64)*
	Chronic sputum production 1.42 (0.96–2.10)
	LCQ total^a^ 1.38 (1.12–1.71)**

*p < 0.05, **p < 0.01, ***p < 0.001

^a^Leicester cough questionnaire total score in reversed tertiles (21.0–16.92, 16.91–14.35 and 14.34–3.00)

^b^Cough duration: (1) < 1 week, (2) 1–3 weeks, (3) 3–8 weeks, (4) 2–12 months, (5) 1–5 years, (6) 5–10 years, (7) > 10 years

## Discussion

We investigated the frequencies and predictors of any (≥ 1) and repeated (≥ 3) doctor’s visits due to cough, among 1109 elderly participants with current cough. Cough-related doctor’s visits during the past year represents the healthcare burden of both current and possible recurrent, separate cough episodes. Doctor’s visits due to current cough represents healthcare burden of a single cough episode, which often had lasted for many years.

Noticeably, over 70% of participants had no cough-related doctor’s visits during the past year, whereas a minority (7.1%) had sought medical help repeatedly. The latter group accounted for most of the yearly doctor’s visits, similarly to younger adults [4]. Thus, healthcare-seeking behaviour due to cough varies greatly from not seeking help at all to heavy healthcare utilisation. The large proportion of non-visitors may be due to the high prevalence of very longstanding (> 5 years) current cough in this population. These participants may have visited a doctor sometime earlier but not during the past year. The percentage of participants with repeated visits during the past year peaked at 1–5 years and decreased after 5 years of cough duration. Thus, the heavy healthcare users due to cough are not those with the longest cough episodes. The proportions of any and repeated visits during the past year were lower in the elderly than in younger Finnish adults (25.9% *vs.* 40.3%, and 7.1% *vs.* 13.8%, respectively) [2]. However, the circumstances of the two surveys were different, as the present one was conducted in the middle of the COVID-19 pandemic.

In the multivariate analysis, current asthma, cough-related quality-of-life impairment, chronic sputum production and impaired self-assessed health status increased the probability of at least one visit during the past year. In turn, the coughers who do not seek medical attention are more likely non-asthmatic, perceive themselves healthier, and their cough is drier and less bothersome in comparison to those who do seek help.

The predictors of repeated doctor’s visits during the past year in the multivariate analysis were bronchiectasis, current asthma, chronic sputum production, and impairment in both self-assessed health status and cough-related quality-of-life. Bronchiectasis was associated with repeated visits in the elderly but not in younger adults [4], which is probably due to bronchiectasis prevalence increasing with age. Asthma, as one of the most common background disorders of cough, predicted repeated visits both in the elderly and in younger adults [4]. As sputum production is associated with both bronchiectasis and asthma [16], chronic phlegmy respiratory diseases seem to increase the cough-related healthcare burden. Both conditions are also characterised by exacerbations which are often treated with prescription drugs, and this may partly explain their associations with repeated visits. The important role of quality-of-life in healthcare use and costs due to cough has been recognised also previously [4, 17, 18]. Routine quality-of-life assessment, as recommended in guidelines [14, 19], may help to detect potential “high-cost patients” due to cough. Of note, duration of current cough was not a risk factor of repeated visits during the past year, inconsistently to younger adults [4]. This is explained by the decrease in visits during the past year towards the most longstanding cough, which was more prevalent in the elderly [2]. Furthermore, the proportions of subjects with repeated visits did not differ significantly between subacute and chronic cough, similarly to younger adults [2]. In this population, the small number of participants with subacute cough decreased the statistical power of these comparisons. However, both studies suggest that recurrent subacute cough episodes, which are often triggered by infections or seasonal allergens, result in similar number of yearly doctor’s visits to those caused by chronic cough.

In this population, 32% of participants had visited a doctor at least once due to current cough. In other community-based studies, the rate of seeking medical care during a single cough episode of any duration varies from 26–40% [2, 20, 21]. Any visit due to current cough became more common as the cough prolonged. However, 60% of participants with current chronic cough had never sought medical help for it. Furthermore, half of those with cough lasting > 10 years remained unevaluated. Thus, cough may prolong unnecessarily when treatable background disorders are missed. Of note, there were more current smokers among non-visitors (2.6%) than among those with any visit (1.1%, *p* = 0.12), or among repeated visitors due to current cough (0%, *p* = 0.04). This may represent doctor avoidance among smokers with cough [21, 22], which is concerning for the diagnostics of potentially life-threatening diseases.

The predictors of any and repeated doctor’s visit due to current cough in the multivariate analyses were cough-related quality-of-life impairment and cough duration. The results seem logical, as the participants have sought to resolve their troublesome and prolonging cough. Doctor-initiated follow-up visits during the diagnostic protocol may also contribute to repeated visits in prolonging current cough to a limited extent. From the background disorders of cough, only asthma was associated with repeated visits due to current cough thus highlighting its socioeconomic impact.

This study has limitations. The response rate was rather low, possibly resulting in selection bias. The impact of smoking on the results may suffer from the underrepresentation of smokers. Also, people with disabilities that affect computer use could not participate. However, the response rate may not affect the main results of repeated doctor’s visits greatly. The doctor’s visits were self-reported which predisposes to recall bias. This probably affected more the results of current cough, since most participants had been coughing for years, but less the results of visits during the past year. Lastly, the ongoing COVID-19 pandemic may have affected both the participants` willingness to seek medical help and the available healthcare resources. This study has strengths also. It was community-based and focused on the elderly. This helps to understand the cough-related healthcare-seeking behaviour in the growing elderly population which is most burdened by chronic cough. This was the first study to characterise separately the healthcare use due to all cough episodes in one year, and due to a single cough episode irrespective of its duration. This study was conducted similarly to our previous study in younger adults, which allows comparison of these two studies.

To conclude, a minority of participants accounted for most of the yearly cough-related doctor’s visits, whereas most participants with chronic cough had never sought medical attention. This may not represent efficient utilisation of the limited healthcare resources. Repeated visitors due to cough were characterised by chronic phlegmy respiratory conditions, and cough-related quality-of-life impairment.
